# Automated verbal autopsy classification: using one-against-all ensemble method and Naïve Bayes classifier

**DOI:** 10.12688/gatesopenres.12891.2

**Published:** 2019-01-23

**Authors:** Syed Shariyar Murtaza, Patrycja Kolpak, Ayse Bener, Prabhat Jha

**Affiliations:** 1 Data Science Lab, Ryerson University, Toronto, Ontario, M5B 2K3, Canada; 2 Centre for Global Health Research, St. Michael's Hospital, Toronto, Ontario, Canada; 3 Dalla Lana School of Public Health, University of Toronto, Toronto, Canada

**Keywords:** COD classification, VA algorithms, CSMF Accuracy, sensitivity, performance assessment

## Abstract

Verbal autopsy (VA) deals with post-mortem surveys about deaths, mostly in low and middle income countries, where the majority of deaths occur at home rather than a hospital, for retrospective assignment of causes of death (COD) and subsequently evidence-based health system strengthening. Automated algorithms for VA COD assignment have been developed and their performance has been assessed against physician and clinical diagnoses. Since the performance of automated classification methods remains low, we aimed to enhance the Naïve Bayes Classifier (NBC) algorithm to produce better ranked COD classifications on 26,766 deaths from four globally diverse VA datasets compared to some of the leading VA classification methods, namely Tariff, InterVA-4, InSilicoVA and NBC. We used a different strategy, by training multiple NBC algorithms using the one-against-all approach (OAA-NBC). To compare performance, we computed the cumulative cause-specific mortality fraction (CSMF) accuracies for population-level agreement from rank one to five COD classifications. To assess individual-level COD assignments, cumulative partially-chance corrected concordance (PCCC) and sensitivity was measured for up to five ranked classifications. Overall results show that OAA-NBC consistently assigns CODs that are the most alike physician and clinical COD assignments compared to some of the leading algorithms based on the cumulative CSMF accuracy, PCCC and sensitivity scores. The results demonstrate that our approach improves the performance of classification (sensitivity) by between 6% and 8% compared with other VA algorithms. Population-level agreements for OAA-NBC and NBC were found to be similar or higher than the other algorithms used in the experiments. Although OAA-NBC still requires improvement for individual-level COD assignment, the one-against-all approach improved its ability to assign CODs that more closely resemble physician or clinical COD classifications compared to some of the other leading VA classifiers.

## Introduction

Verbal autopsy (VA) is increasingly being used in developing countries where most deaths occur at home rather than in hospitals, and causes of death (COD) information remains unknown ^1^. This gap in information prevents evidence-based healthcare programming and policy reform needed to reduce the global burden of diseases ^2^. VA consists of a structured questionnaire to gather information on symptoms and risk factors leading up to death from family members of the deceased. Each completed survey is then typically reviewed independently by two physicians, and COD assignment is done using World Health Organization (WHO) International Classification of Disease (ICD) codes ^3^. If there is disagreement in assignment, then the VA undergoes further review by a senior physician ^4, 5^. Efforts are underway to make verbal autopsies the part of the civil registration system of countries to ensure that effective policies can be developed to prevent global diseases ^6^.

In recent years, efforts have been made to automate VA COD assignment using various computational algorithms in an attempt to further standardize VA COD assignment and alleviate physician time and costs ^7– 14^. The current leading computational VA techniques include, InterVA-4 ^8^, Tariff ^7^, InSilicoVA ^9^, King-Lu ^11^, and Naïve Bayes Classifier (NBC) ^12^. InterVA-4 employs medical-expert-defined static weights for symptoms and risk factors given a particular COD, and subsequently calculates the sum of these weights to determine the most likely COD ^8^. Conversely, Tariff was pre-trained on the Population Health Metrics Research Consortium (PHMRC) VA data to compute tariffs, which express the strength of association between symptoms and CODs that are later summed and ranked to determine a COD; the same procedure is used on the test dataset, with the resultant summed and ranked tariffs scores compared against the pre-trained COD rankings ^15^. InSilicoVA assigns CODs by employing a hierarchical Bayesian framework with a naïve Bayes calculation component; it also computes the uncertainty for individual CODs and population-level COD distributions ^9^. The King-Lu method measures the distribution of the COD and symptoms in the VA training dataset and uses these to predict CODs in the VA test dataset ^11^. Lastly, NBC predicts the COD after computing the conditional probabilities of observing a symptom for a given COD from the VA training dataset, and then applying the Bayes rule against these probabilities ^12^. These existing automated classification algorithms, however, generate low predictive accuracy when compared against physician VA or hospital-based COD diagnoses ^9, 12, 16, 17^. Leitao *et al*. ^18^ in their systematic review of automated verbal autopsy classification algorithms concluded that there is need to improve automated classification techniques to enable wider and more reliable employment in the field.

The aim of our research is also a classification method for predicting CODs using responses from structured questions in a VA survey. We used a different strategy by training multiple NBC algorithms ^19^ using the one-against-all approach (OAA-NBC) ^20, 21^. We have chosen NBC algorithm and one-against-all ensemble method of machine learning because former has shown better results on VA surveys ^12^ and later has shown better results in machine learning literature ^20, 21^. OAA-NBC generates ranked assignments of CODs for 26,766 deaths from four globally diverse VA datasets (one VA dataset was divided into four datasets; a total of seven datasets were used for analysis). We also compare our technique against the current leading algorithms Tariff ^7^, InterVA-4 ^8^, NBC ^12^ and InSilicoVA ^9^ on the same deaths used for OAA-NBC.

## Methods

### Datasets

In order to test the performance of the algorithms, we used four main datasets, containing information on a total of 26,766 deaths: three physician COD diagnosed VA datasets, namely the Indian Million Death Study (MDS) ^22^, South African Agincourt Demographic Surveillance Sites (HDSS) dataset ^23^, and Bangladeshi Matlab HDSS dataset ^24^, and one health facility diagnosed COD dataset, namely the PHMRC VA data collected from six sites in four countries (India, Mexico, the Philippines and Tanzania) ^25, 26^. We used four combinations of the PHMRC data by age group (adult and child) and by site (all versus India-only); this filtering was done to determine the effect on results when deaths were collected from the same geographical setting. A total of seven datasets were used and are summarized in Table 1. These datasets are publicly available, except for the MDS, and have been used in other studies ^12, 16, 26^.

**Table 1.** Verbal autopsy (VA) datasets used in the study *.

	MDS	Agincourt	Matlab	PHMRC- Adult (All Sites)	PHMRC- Child (All Sites)	PHMRC- Adult (India)	PHMRC- Child (India)
Region	India	South Africa	Bangladesh	Multiple ^1^	Multiple	Andhra Pradesh and Uttar Pradesh	Andhra Pradesh and Uttar Pradesh
# of deaths	12,225	5,823	2,000	4,654	2,064	1233	948
Ages	1–59 months	15–64 years	20–64 years	12–69 years	28 days– 11 years	12–69 years	28 days– 11 years
# of grouped CODs	15	16	15	13	9	13	9
# of Symptoms	90	88	214	224	133	224	133
Physician Classification	Dual physician agreement	Dual physician agreement	Two level physician classification	Hospital certified cause of death, including clinical and diagnostic tests	Hospital certified cause of death, including clinical and diagnostic tests	Hospital certified cause of death, including clinical and diagnostic tests	Hospital certified cause of death, including clinical and diagnostic tests

^1^Six sites in total: Andhra Pradesh and Uttar Pradesh (India), Distrito Federal (Mexico), Bohol (Philippines) and Dar es Salaam and Pemba (Tanzania); applicable to both adult and child age group specific datasets.

*MDS, Agincourt and Matlab had CODs assigned by physician review of VA datasets and PHMRC is based on physician review of clinical diagnostic criteria

The MDS VA dataset used in this study contained information on 12,225 child deaths from ages one to 59 months. For each death, two trained physicians independently and anonymously assigned a WHO ICD version 10 code ^27^. In the cases where the two physicians did not initially agree or reconcile on a COD, a third senior physician adjudicated ^22^. Similarly, the Agincourt dataset ^23^ underwent dual physician COD assignment on its 5,823 deaths for ages 15 to 64 years. COD assignment was slightly different for the Matlab dataset which had 2,000 deaths for ages 20 to 64 years; a single physician assigned a COD, followed by review and verification by a second physician or an experienced paramedic ^24^. In contrast, the PHMRC dataset was comprised of 6,718 hospital deaths that were assigned a COD based on certain clinical diagnostic criteria, including laboratory, pathology, and medical imaging findings ^25, 26^. For each VA dataset, we grouped the physician assigned CODs into 17 broad categories, refer to Table 2. We also show the distribution of records for each COD for each of the seven datasets used in our study.

**Table 2.** Cause of death (COD) list with absolute death counts by VA dataset *.

Groups	Causes	Agincourt	Matlab	MDS	PHMRC All Sites Adult	PHMRC Indian Adults	PHMRC All Sites Children	PHMRC Indian Children
1	Acute respiratory	110	11	3392	304	81	532	141
2	HIV/AIDS	2012	NA	5	NA	NA	NA	NA
3	Diarrhoeal	66	29	2711	101	41	`256	112
4	Pulmonary TB	690	43	78	177	21	NA	
5	Other and unspecified infections	432	79	2514	622	174	376	187
6	Neoplasms (cancer)	244	352	96	497	19	28	15
7	Nutrition and endocrine	70	90	372	NA	NA	NA	NA
8	Cardiovascular Diseases	381	714	18	928	242	76	25
9	Chronic Respiratory	27	129	21	84	52	NA	NA
10	Liver cirrhosis	89	100	112	234	59	NA	NA
11	Other non- communicable diseases	221	244	1345	697	125	186	80
12	Neonatal conditions	NA	NA	410	NA	NA	NA	NA
13	Road and transport injuries	219	49	95	124	32	92	64
14	Other injuries	366	68	659	471	218	324	259
15	Ill-defined	711	35	397	NA	NA	194	65
16	Suicide	125	34	NA	70	33	NA	NA
17	Maternal	60	23	NA	345	136	NA	NA

*MDS, Agingcourt and Matlab had CODs assigned by physician review of VA datasets and PHMRC is based on physician review of clinical diagnostic criteria.

### One-against-all Naïve Bayes (OAA-NBC) approach

**Figure 1.**
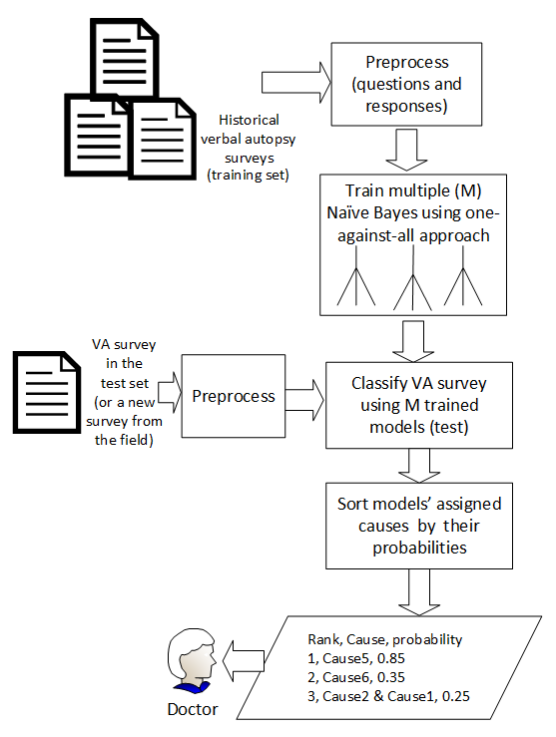
Overview of one-against-all approach.

An overview of our approach is shown in Figure 1. We transformed each VA dataset into binary format with VA survey questions being the attributes (columns), answers being the values of cells in rows (re-coded into binary format with ‘Yes’ coded as 1 and ‘No’ as 0) and CODs (group number identifier listed in Table 2) being the last (or the first) column. For all VA datasets, a death was represented as a row (record).

We divided each VA dataset into training and testing datasets. We trained multiple NBC models ^19^ on the transformed training datasets using the one-against-all approach ^20, 21^. We chose NBC because it showed better results on VA surveys in the past ^12^. The one-against-all approach was used because it improves the algorithm’s classification accuracy on datasets with several categories of dependent variables as demonstrated by past literature ^20, 21^. This will be explained in detail in the next section. During testing, the trained NBC models assigned CODs to each death in the testing dataset. The assigned causes were ordered by their probabilities with the assumption that top cause would most likely be the real cause.

***Training Naïve Bayes using one-against-all approach.*** NBC uses a training dataset to learn the probabilities of symptoms and their CODs ^12, 19^. NBC first measures the probability of each COD, P(COD), in the training dataset. Secondly, it determines the conditional probabilities of each symptom given a particular COD, P(Sym|COD). Thirdly, NBC determines the probability of every COD given a VA record in the test set, i.e., P(COD|VA).

$$\text{P(COD|}\;\text{VA)}=\text{P(COD)}\;\prod_{Sym\;\in \;VA}P(Sym|COD)$$

**Equation 1.** Conditional probability of COD given a VA record.

P(COD|VA) is determined by taking the product of all P(Sym|COD) (i.e., all symptoms in the VA record) and P(COD). The highest P(COD|VA) value determines that COD as the correct COD. In particular, we chose the Naïve Bayes Multinomial classification algorithm that estimates probabilities by using a maximum likelihood estimate which is readily available in data mining software applications like Weka ^19, 20^.

$${\text{COD}}_{\text{NBC}}\text{=}argmax_{COD\;\in CODs}\text{P}(\mathrm{COD}|\text{VA})$$

**Equation 2.** Select the class with the maximum probability.

**Figure 2.**
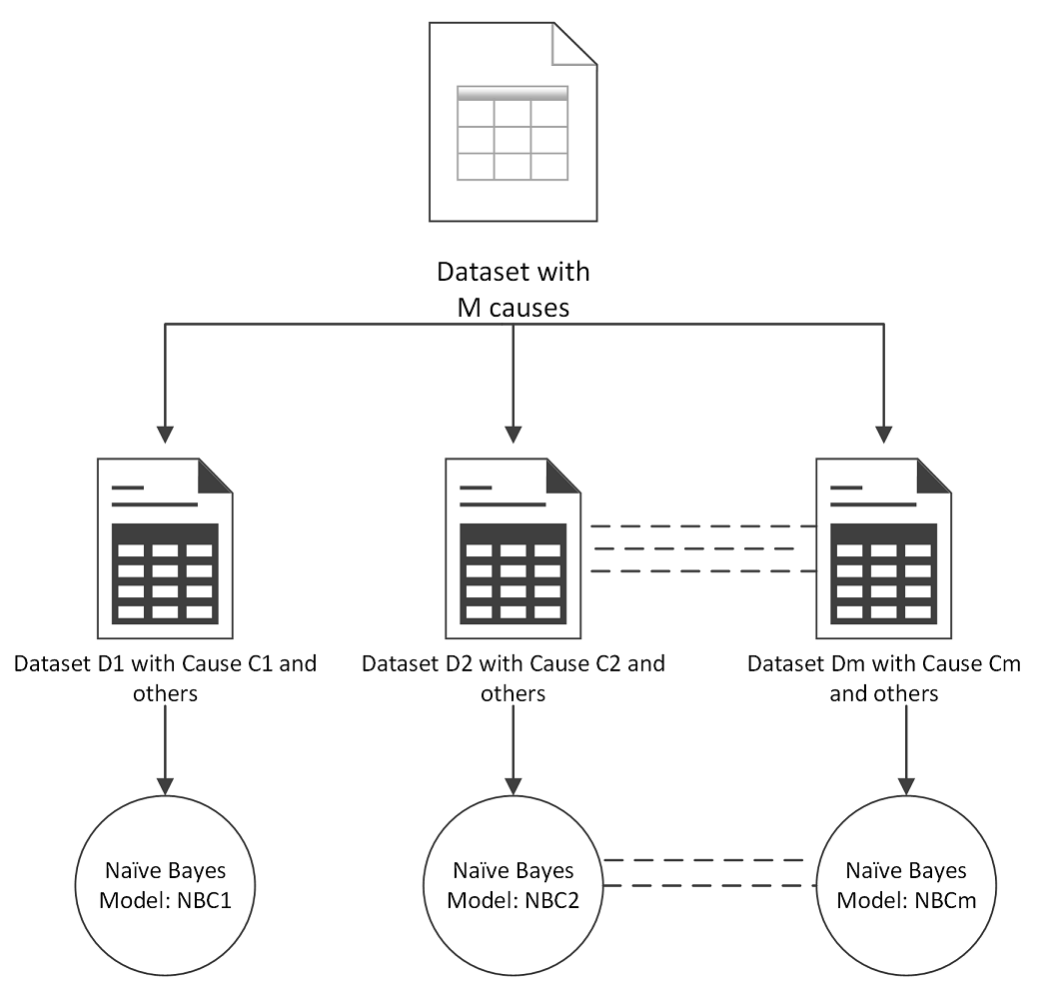
One-against-all approach for ensemble learning.

In the one-against-all approach, we built an NBC model for each COD instead of one model for all CODs. In this approach, a dataset with M categories of CODs (dependent variables) was decomposed into M datasets with binary categories (CODs). Each binary dataset Di had a COD Ci (where i = 1 to M) labelled as positive and all other CODs labelled as negative with no two datasets having the same CODs labelled as positive. Finally, NBC was trained on each dataset Di resulting in M Naïve Bayes models, as shown in Figure 2. Each model was then used to classify the CODs for records in the test dataset producing a probability of classification. The cause Ci (where i=1 to M) with the highest probability was considered as the correct classification.

***Testing OAA-NBC on new surveys.*** During testing, each Naïve Bayes model predicted a COD for each VA record in the test dataset, resulting in a list of CODs for each VA record in the test dataset. The list of assigned CODs is sorted by the COD probabilities. We made a minor modification in the one-against-all approach; instead of selecting a COD with the highest probability, we ranked the CODs in descending order of their probabilities for each VA record. We kept the ranked probabilities to generate cumulative performance measures, which are described in detail in the next section.

### Assessment methods

A VA algorithm’s performance is measured by quantifying the similarity between the algorithm’s COD assignments to physician review (or clinical diagnoses in PHMRC) assignments. Since the community VA datasets included in this study come from countries that have weak civil and death registration, physician review is the most practical and relatively accurate (and only) option to use for assessing algorithm performance. Moreover, given that these deaths are unattended, it follows that there is no ‘gold standard’ for such community VA datasets. Nevertheless, we are confident in the robustness of dual physician review as initial physician agreement (i.e. where two physicians agreed right at the onset of COD coding) was relatively high; e.g., 79% for MDS and 77% for Agincourt.

We measured and compared the individual and population-level performance of all of the algorithms using the following metrics: sensitivity, partially chance corrected concordance (PCCC) and cause-specific mortality fraction (CSMF) accuracy. These measures are commonly used in VA studies ^12, 16, 28^. They are shown in Equation 3 – Equation 5. They are helpful in objectively assessing the performance of VA algorithms, as they provide a robust strategy to assess an algorithm’s classification ability for test datasets with widely varying COD distributions ^13, 28^.

$$Sensitivity=\frac{True\;positive}{True\;positive+False\text{​}\;Negative}$$

**Equation 3.** Sensitivity of classification

$$PCCC(k)=\frac{S-\frac{k}{n}}{1-\frac{k}{n}}$$

$$\text{Where}\;\text{S}=\frac{True\;positive}{True\;positive+False\text{​}\;Negative}$$

**Equation 4.** Partially chance corrected concordance (PCCC) of classification: S is the fraction of positively (correctly) assigned causes when the correct cause is in the top k assigned causes out of total n causes.

Sensitivity and PCCC are metrics that assess the performance of an algorithm for correctly classifying the CODs at the individual level. Sensitivity measures the proportion of death records that are correctly assigned for each COD ^13^. Similarly, PCCC computes how well a VA classification algorithm classifies the CODs at the individual-level while also taking chance (likelihood that it was randomly assigned a COD) into consideration ^9, 12, 13, 16^.

$$CSMF\;Accuracy=1-\frac{\sum_{j=1}^{n}\left|CSMF_{j}^{True}-CSMF_{j}^{Pred}\right|}{2(1-Minimum\left(CSMF_{j}^{True}\right))}$$

$$\text{Where}\;CSMF^{Pred}=\frac{\left(TP+FP\right)}{N}\;and\;CSMF^{True}=\frac{\left(TP+FN\right)}{N}$$

**Equation 5.** Cause-specific mortality fraction (CSMF) Accuracy of classification: n is the total COD and N is the total records.

In contrast, CSMF accuracy is a measure for assessing how closely the algorithms classified the overall COD distribution at the population level ^13^. It can be observed from Equation 5 that CSMF accuracy computes the absolute error between predicted COD distributions by an algorithm (pred) and the observed (true) COD distributions.

We measured the cumulative values of sensitivity, PCCC and CSMF accuracy on each rank and for each algorithm; e.g., sensitivity at rank two represented the sensitivity of both rank one and rank two classifications, which facilitated in measuring the overall performance of the algorithms for classifications at the top two or more ranks. For example, if sensitivity value was 60% at rank one and sensitivity value was 15% at rank two for a method, then the cumulative sensitivity was 75% at rank 2. The use of cumulative values for reporting results is common in applied machine learning literature (e.g., see Murtaza *et al*. ^29^ and Wong *et al*. ^30^). It only adds additional information to the traditional way of reporting results (which are only about rank 1) and useful when there are multiple classes (causes of deaths). Finally, we also performed a statistical test of significance on the results of all the datasets to ascertain that the difference in results was not by chance. The statistical test depends on the data distribution and association between experiments. We used Wilcoxon signed rank test as we were unsure about normal data distribution of our results. Our null hypothesis was that there was no significant difference between OAA-NBC and another algorithm. This is further discussed in the results section.

### Experimental setup

In order to compare the performance between OAA-NBC, InterVA-4 ^8^, Tariff ^7^, InSilicoVA ^9^ and NBC ^12^, we follow a seven step procedure. In Step one, we partitioned each VA dataset using the commonly used evaluation criteria in data mining: 10-fold cross validation ^20^. In 10-fold cross validation, a dataset was divided into 10 parts. Each part was created by using stratified sampling method—i.e., each part contained the same proportion of standardized CODs as the original dataset. In Step two, we selected one part for testing and nine parts for training from each VA dataset. In Step three, we trained OAA-NBC, InterVA-4, Tariff, InSilicoVA and NBC on the designated training data subsets from each partitioned VA dataset. In Step four, we generated classifications with ranks for each algorithm on the test part per VA dataset. In Step five, we calculated the cumulative sensitivity, PCCC and CSMF accuracy for each rank per each VA dataset. In Step six, we repeated the process from Step two to Step five up to 10 repetitions with a different part for testing in each turn and for each VA dataset. In Step seven, we computed the mean sensitivity, PCCC and CSMF accuracy for each rank per VA dataset and algorithm.

We implemented OAA-NBC in Java and with Weka API ^20^. Weka provides APIs for one-against-all approach and Naïve Bayes Multinomial classifier ^20^. We used the OpenVA package version 1.0.2 in R to implement InterVA-4, Tariff, InSilicoVA and NBC algorithms. The data format also was transformed into InterVA-4 input format (Y for 1 and empty for 0 values). It is important to note that the Tariff version provided in the OpenVA package is computationally different from the IHME’s SmartVA-Analyze application tool. We used custom training option for InterVA-4 and InSilicoVA as present in OpenVA package in R. In custom training, the names of symptoms do not need to be in the WHO standardised format, and the rankings of the conditional probability P(symptom|cause) are determined by matching the same quantile distributions in the default InterVA P(symptom|cause). The reason for choosing customized training instead of using pre-trained global models is that different datasets have different proportions of symptoms and causes of deaths, and custom training allows algorithms to generate models customized for the dataset. It also allows for fair evaluation across algorithms, especially for the ones that only work by using customized training on datasets and acquire more knowledge of the dataset during testing.

We performed data partitioning, as discussed in Step 1, using Java and Weka’s ^20^ stratified sampling API. Each algorithm was executed on that partitioned data. We used a separate Java program to compute the cumulative measures of sensitivity, PCCC and CSMF accuracy on the COD assignments of each algorithm for each VA dataset. This process ascertained that our evaluation measures were calculated in the exact same manner. Our source code for all the experiments is available on GitHub and is archived at Zenodo ^31^.

## Results

### Ranked CSMF accuracy comparison

**Figure 3.**
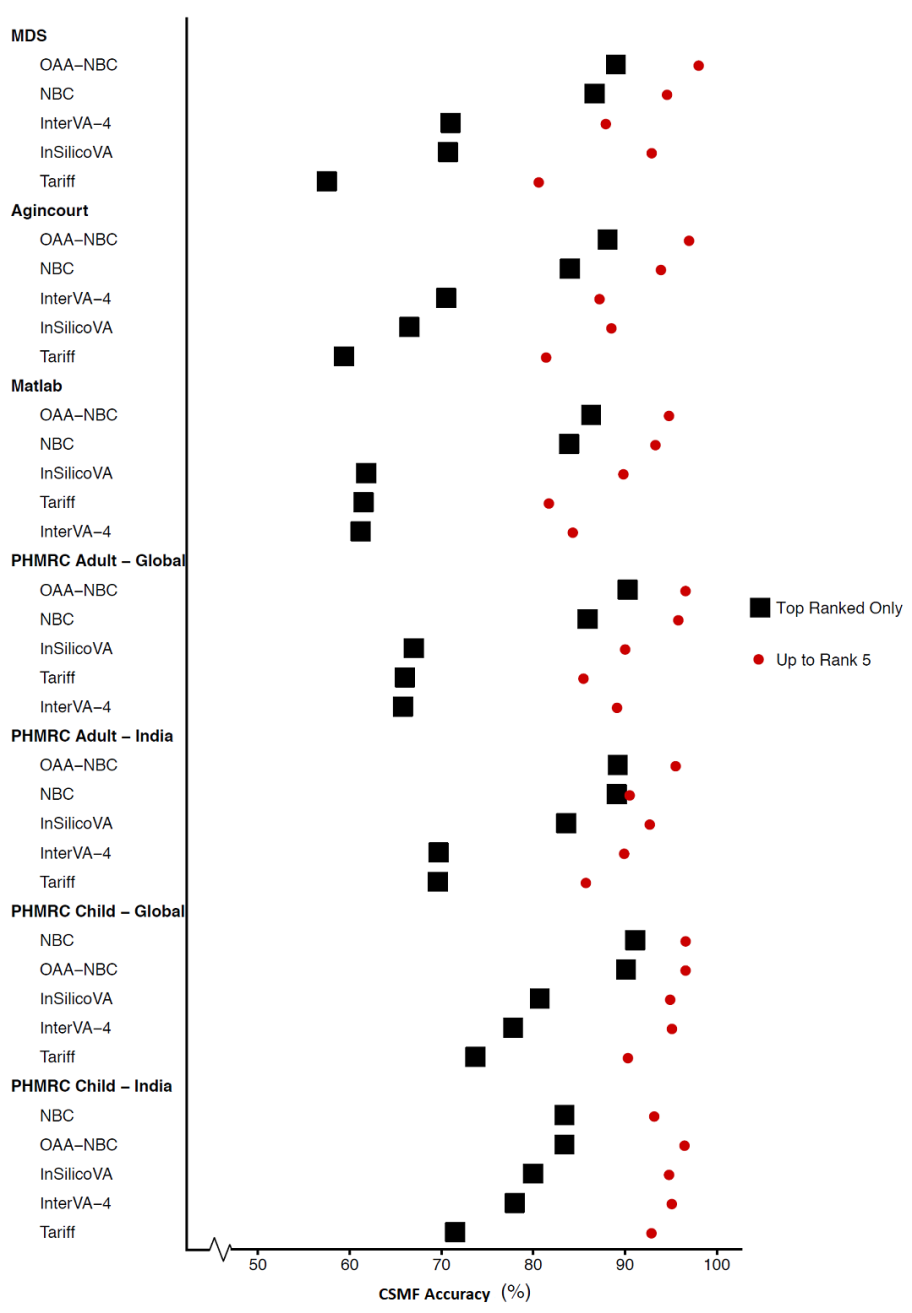
Ranks 1 and 5 cause-specific mortality fraction (CSMF) accuracies (agreement) across VA datasets and algorithms.

Figure 3 shows the mean CSMF accuracy values by algorithms across all VA datasets using rank one (most likely) cause (COD) assignments and the fifth most likely cause assignments (rank five). Note that the fifth rank shows the cumulative CSMF accuracy values from rank 1 to rank 5 as described earlier. OAA-NBC produced the highest CSMF accuracy values for most of the VA datasets, ranging from 86% to 90% for rank one; it came second or identical to NBC for the PHMRC child datasets (global and India). Furthermore, CSMF accuracy values for OAA-NBC were relatively consistent across the VA datasets compared to some of the other algorithms that varied considerably, such as Tariff, InterVA-4 and InSilicoVA. As expected, the cumulative CSMF accuracy values increased the overall CSMF accuracy values for each algorithm when including the top five ranked classifications for every VA dataset.

### Ranked sensitivity comparison

Individual-level cumulative sensitivity results for classification ranks one and five are shown in Table 3; cumulative PCCC values are not shown as the values were very close to the cumulative sensitivity values. It can be observed from Table 3 that OAA-NBC got the highest sensitivity values for the first ranked (most likely) COD assignments compared to the other algorithms, ranging between 53–63%. When considering all top five ranked classifications, OAA-NBC improved sensitivity values by 31–38%, with cumulative values ranging from 91–95%. In the case of Tariff, InterVA-4 and InSilicoVA, the sensitivity values were significantly lower (10–40%) in comparison to OAA-NBC; NBC did not differ substantially from OAA-NBC, as differences only range from 3–7%. These results show that OAA-NBC consistently yields closer agreement with the physician review or clinical diagnoses at the individual-level than the other algorithms on most of the VA datasets.

**Table 3.** Cumulative sensitivity of rank 1 and rank 5 (1-5) for COD (cause of death) classifications by VA (Verbal Autopsy) datasets and algorithms.

	VA dataset, rank, cumulative sensitivity (%)													
	MDS		Matlab		Agincourt		PHMRC Adult -Global		PHMRC Adult - India		PHMRC Child - Global		PHMRC Child - India	
Algorithm	Rank 1	Rank 1-5	Rank 1	Rank 1-5	Rank 1	Rank 1-5	Rank 1	Rank 1-5	Rank 1	Rank 1-5	Rank 1	Rank 1-5	Rank 1	Rank 1-5
Tariff	31.5	71.4	40.7	75.3	27.5	72.1	35.9	74.7	44.0	79.4	37.0	83.7	39.5	86.3
InterVA-4	48.8	82.7	34.8	79.3	46.3	78.8	36.3	82.2	41.1	84.6	45.1	91.8	51.2	93.0
InSilicoVA	45.6	85.9	35.6	80.8	35.8	80.3	35.0	79.5	50.3	87.3	43.3	89.6	49.4	92.4
NBC	56.0	90.1	50.7	87.2	48.2	87.4	47.7	88.1	54.8	86.1	51.5	93.1	58.6	92.4
OAA-NBC	61.1	94.3	57.9	91.2	55.5	93.1	53.1	91.0	60.1	93.1	54.6	93.4	63.0	94.7

We also performed a Wilcoxon signed rank statistical test on the reported sensitivity values in Table 3, generated from the five algorithms (we also included rank two to rank four values which are not shown in the table to minimize space but present in Supplementary file 1). For 35 observations (five ranks and seven data sets), the Wilcoxon signed ranked test yielded Z-score=5.194 and two tailed p-value=2.47 x 10 ^-7 ^ between OAA-NBC and NBC. It yielded the same Z-scores and two tailed p-values against InSilicoVA, InterVA-4, and Tariff. Thus, this showed statistically significant differences between the sensitivity values generated by OAA-NBC and the four other algorithms (p < 0.05). Similarly, we conducted the Wilcoxon signed rank test on 35 observations of CSMF accuracy values for the five different algorithms. The Z-score is 4.248 and p-value is 2.15 x 10 ^-5^ between OAA-NBC and NBC. The same Z-score and p-value were also obtained for the tests between OAA-NBC and other algorithms: InSilicoVA, InterVA-4, and Tariff. We found statistically significant differences between OAA-NBC and the other algorithms in all the comparisons.

Thus, the use of one-against-all approach with NBC (OAA-NBC) improved the performance of COD classification for VA records, and yielded better COD assignments at the population- and individual-level, which were statistically different and not attributed to chance compared to the four other algorithms. This also conformed to the machine learning literature that the one-against-all approach improved the performance of classification algorithms when there were more than two classes (CODs) ^21^. However, this did not indicate that OAA-NBC did not require improvement, as the overall sensitivity for the top ranked CODs per VA record was still lower than 80%. We also made an additional assessment on the COD sensitivity. Table 4 shows the sensitivity per cause for first ranked predictions and VA dataset for each algorithm (PHMRC Indian datasets are excluded as their results are similar to PHMRC global datasets and this minimizes space too). The sensitivity values varied per VA dataset and cause for all of the algorithms; road and transport injuries and other injuries were the only causes that OAA-NBC predicted consistently well for four out of the five VA datasets. However, there were several causes where the sensitivity of the classifications by OAA-NBC were lower than 50%, and in some cases, 0% (four causes in MDS and two causes in PHMRC – Child global datasets). Sensitivity values were 0% for COD groups that have proportion of records near 1% per VA dataset (number of records for each COD in VA datasets are shown in Table 2). In general, the algorithms performances varied on different CODs for certain conditions in VA datasets. For example, classifications were equal to or under 10% across all algorithms for HIV/AIDs, cancers, cardiovascular disease, and chronic respiratory diseases in the MDS dataset. Algorithms like OAA-NBC and NBC mostly had better sensitivity for COD groups that had higher proportion of records in training dataset. However, this was not always the case, and better sensitivity values also depended on how distinguishable VA records of a COD group were from all other COD groups. In the next section, we discuss the problem and effects of imbalance within datasets on the algorithms’ classification accuracy.

**Table 4.** Top ranked (most likely) sensitivity scores per COD (cause of death) by VA (verbal autopsy) dataset and algorithm with physician assigned COD distributions.

		Cause, sensitivity (%)																
VA Dataset	Algorithm	Acute respiratory	HIV/AIDS	Diarrhoeal	Tuberculosis	Other & unspecified infections	Cancers	Nutrition & endocrine	Cardiovascular diseases	Chronic Respiratory	Liver cirrhosis	Other NCDs	Neonatal conditions	Road & transport injuries	Other injuries	Ill-defined	Suicide	Maternal
MDS	Physician *	***27.7***	***0.04***	***22.2***	***0.6***	***20.6***	***0.8***	***3.0***	***0.1***	***0.2***	***0.9***	***11.0***	***3.3***	***0.8***	***5.4***	***3.2***	***-***	***-***
	Tariff	36.1	10.0	47.5	42.5	19.7	16.7	31.7	5.0	0.0	23.9	3.0	11.2	84.3	57.3	25.7	-	-
	InterVA-4	78.0	0.0	55.3	51.0	43.6	9.5	0.9	8.3	3.3	29.2	1.0	15.4	70.7	71.5	10.4	-	-
	InSilicoVA	61.5	0.0	55.7	50.0	32.4	0.6	42.3	0.0	0.0	21.0	6.8	13.9	82.1	69.6	63.3	-	-
	NBC	74.9	0.0	70.4	31.6	46.5	4.1	41.3	0.0	1.7	18.0	22.6	15.2	73.0	80.1	49.0	-	-
	OAA-NBC	85.2	0.0	78.5	17.9	51.5	0.0	25.3	0.0	0.0	4.5	23.0	11.0	79.8	80.6	25.7	-	-
Matlab	Physician *	***0.5***	***-***	***1.4***	***2.1***	***3.9***	***17.6***	***4.5***	***35.7***	***6.4***	***5.0***	***12.2***	***-***	***2.4***	***3.4***	***1.7***	***1.7***	***1.1***
	Tariff	15.0	-	53.3	55.0	15.0	41.0	61.1	38.1	79.8	50.0	9.9	-	57.0	51.2	13.3	70.8	16.7
	InterVA-4	0.0	-	26.7	51.0	29.8	48.6	21.1	32.1	42.6	61.0	7.4	-	81.5	37.1	0.0	70.8	15.0
	InSilicoVA	20.0	-	50.0	34.5	11.4	17.1	34.4	47.9	71.3	53.0	8.2	-	91.5	19.0	13.3	86.7	8.3
	NBC	10.0	-	21.7	42.5	15.4	55.4	43.3	64.1	66.5	57.0	20.0	-	83.5	15.0	21.7	76.7	5.0
	OAA-NBC	20.0	-	51.7	30.5	7.5	67.6	38.9	75.3	75.8	53.0	23.8	-	96.0	39.5	2.5	75.8	5.0
Agincourt	Physician *	***1.9***	***34.5***	***1.1***	***11.8***	***7.4***	***4.2***	***1.2***	***6.5***	***0.5***	***1.5***	***3.8***	***-***	***3.8***	***6.3***	***12.2***	***2.1***	***1.0***
	Tariff	44.3	21.4	39.8	53.3	7.2	24.6	69.3	24.7	30.8	50.0	19.6	-	80.8	41.0	3.0	14.0	60.3
	InterVA-4	36.1	74.5	34.7	59.9	12.5	28.1	25.8	13.7	43.3	50.7	9.9	-	78.4	64.7	0.0	21.9	29.2
	InSilicoVA	53.1	29.3	31.2	60.9	11.4	26.2	32.8	14.8	35.8	41.4	18.5	-	81.5	52.7	29.7	79.8	52.1
	NBC	41.2	59.4	27.9	60.8	26.6	35.3	33.2	28.3	33.3	39.1	16.6	-	79.3	63.3	27.1	69.2	53.3
	OAA-NBC	39.1	77.9	24.3	48.0	52.3	28.7	42.9	44.1	3.3	35.8	19.0	-	82.6	82.0	26.7	6.4	48.3
PHMRC - Adult Global	Physician *	***6.5***	***-***	***2.2***	***3.8***	***13.4***	***10.7***	***-***	***19.9***	***1.8***	***5.0***	***15.0***	***-***	***2.7***	***10.1***	***-***	***1.5***	***7.4***
	Tariff	26.0	-	28.6	47.4	26.8	48.7	-	30.3	19.3	64.0	5.8	-	64.0	37.8	-	22.9	89.9
	InterVA-4	14.5	-	5.9	14.6	45.8	47.7	-	32.6	45.4	87.2	13.0	-	29.2	40.8	-	25.7	61.8
	InSilicoVA	16.1	-	36.7	22.6	27.6	39.4	-	25.2	32.1	46.9	13.1	-	76.1	59.0	-	35.7	80.3
	NBC	26.7	-	31.7	30.0	40.7	60.0	-	49.4	41.7	60.6	21.3	-	61.4	69.6	-	35.7	84.1
	OAA-NBC	22.7	-	22.8	20.3	52.1	64.2	-	64.6	27.4	62.4	26.3	-	59.7	74.3	-	18.6	90.1
PHMRC - Child Global	Physician *	***25.8***	***-***	***12.4***	***-***	***18.2***	***1.4***	***-***	***3.7***	***-***	***-***	***9.0***	***-***	***4.5***	***15.7***	***9.4***	***-***	***-***
	Tariff	28.9	-	56.2	-	20.5	6.7	-	14.5	-	-	22.7	-	67.8	62.2	36.4	-	-
	InterVA-4	69.9	-	45.3	-	25.8	43.3	-	5.0	-	-	8.6	-	78.4	63.5	18.4	-	-
	InSilicoVA	39.3	-	45.6	-	26.9	35.0	-	10.4	-	-	17.2	-	87.1	86.4	29.2	-	-
	NBC	60.5	-	48.4	-	45.5	10.0	-	15.7	-	-	12.9	-	83.9	85.5	27.2	-	-
	OAA-NBC	71.0	-	53.4	-	46.6	0.0	-	0.0	-	-	8.5	-	90.4	91.0	23.0	-	-

*Proportion of deaths assigned for each COD by physician(s) review of VA datasets (MDS, Agincourt and Matlab) or by physician’s clinical diagnoses (PHMRC).

## Discussion

Our approach (OAA-NBC) produces better population and individual-level agreement (sensitivity) from different VA surveys compared to other algorithms. However, the overall sensitivity values are still in the range of 55–61% and not greater than 80% for the top ranked COD assignments. There are several reasons for the low sensitivity values; firstly, each VA dataset is unique, with varying amounts of overlapping or different symptoms. In this respect, the symptom-cause information (SCI) is unique to each VA dataset, and so, some of the algorithms could have had more trouble generating adequate SCIs due to the logic employed by the algorithm itself and VA data. This could help explain the low sensitivity scores by cause and per algorithm for the MDS data, which is one of the VA datasets with the fewest amounts of symptoms, and which could have impacted the SCI used for COD assignment by the algorithms. Conversely, some algorithms like InterVA-4 (when you specify the format as following the WHO 2012 or 2014 VA Instrument) require a set of predefined symptoms, or else prefer independent symptoms (i.e. had a fever) over dependent symptoms (i.e. fever lasted for a week) or interdependent symptoms (i.e. did s/he have diarrhoea and dysentery); the absence of such symptoms would also impact the algorithms’ ability to classify VA records correctly. A solution to this problem is to have better differentiating symptoms for each COD.

One may argue that algorithms, such as InterVA-4 and InSilicoVA (non-training option), which use a different input, namely symptom list, based on WHO’s forms for assigning CODs and do not need training on data, would be unfairly evaluated by using customized training. We converted symptoms in our datasets to WHO standardised names and evaluated InterVA-4, and InSilicoVA on the datasets. We used the same method of 10-fold cross validation method as we used in our experiments earlier but we only provided a test set for each fold to the algorithms for assigning causes of deaths based on standardised symptom names. The output of these algorithms was one of the 63 standardised CODs. We mapped these 63 causes to our 17 CODs for a fair evaluation (see Table 6 for complete details on mapping to the 17 COD categories). We observed that sensitivity for rank one for InterVA-4 remained between approximately 25% and 42%, and sensitivity for InSilicoVA remained between 20% and 43% on all datasets. The use of pre-trained models on standardized VA data inputs did not yield any better results than customized training on datasets.

**Table 5.** Comparison of cumulative sensitivity and cause-specific mortality fraction (CSMF) accuracy of rank 1 and 5 classifications using Dirichlet distribution on MDS and Matlab data.

Algorithm	VA dataset, rank, cumulative sensitivity and CSMF accuracy (%)							
	MDS				Matlab			
	Sensitivity		CSMF accuracy		Sensitivity		CSMF accuracy	
	Rank 1	Rank 1-5	Rank 1	Rank 1-5	Rank 1	Rank 1-5	Rank 1	Rank 1-5
Tariff	29.0	64.7	53.7	74.6	45.2	79.0	54.6	80.8
InterVA-4	33.6	63.9	49.4	70.7	33.4	71.5	51.6	75.1
InSilicoVA	38.1	75.9	57.2	80.5	37.7	81.4	59.4	85.8
NBC	41.7	74.7	60.4	79.6	38.7	73.7	57.6	76.7
OAA-NBC	41.0	75.0	59.8	79.2	45.6	86.2	60.4	88.3

**Table 6.** Complete mapping of ICD-10 (international classification of diseases 10 ^th^ revision) and WHO (World Health Organization) cause labels to the cause list used for performance assessments.

No.	Cause of Death	WHO list of Causes	ICD-10 Range
1	Acute respiratory	Acute resp infect incl pneumonia, Neonatal pneumonia	H65-H68, H70-H71, J00-J22, J32, J36, J85-J86, P23
2	HIV/AIDS	HIV/AIDS related deaths	B20-B24
3	Diarrhoeal	Diarrhoeal diseases	A00-A09
4	Pulmonary TB	Pulmonary tuberculosis	A15-A16, B90, J65
5	Other and unspecified infections	Sepsis (non-obstetric), Malaria, Measles, Meningitis and encephalitis, Tetanus, Pertussis, Haemorrhagic fever, Other and unspecified infect dis, Neonatal sepsis	A17-A33, A35-A99, B00-B17, B19, B25-B89, B91-B99, C46, D64, D84, G00-G09, H10, H60, I30, I32-I33, K02, K04-K05, K61, K65, K67, K81, L00-L04, L08, M00-M01, M60, M86, N10, N30, N34, N41, N49, N61, N70-N74, P35- P39, R50, R75, ZZ21
6	Neoplasms (cancer)	Oral neoplasms, Digestive neoplasms, Respiratory neoplasms, Breast neoplasms, Reproductive neoplasms MF, Other and unspecified neoplasms	C00-C26, C30-C45, C47-C58, C60-C97, D00-D48, D91, N60, N62-N64, N87, R59
7	Nutrition and endocrine	Severe anaemia, Severe malnutrition	D50-D53, E00-E02, E40-E46, E50-E64, X53-X54
8	Cardiovascular Diseases (CVD)	Diabetes mellitus, Acute cardiac disease, Stroke, Other and unspecified cardiac dis	E10-E14, G45-G46, G81-G83, I60-I69, I00-I03, I05-I15, I26-28, I31, I34-I52, I70-I99, R00-R01, R03, ZZ23
9	Chronic respiratory	Chronic obstructive pulmonary dis, Asthma	J30-J31, J33-J35, J37-J64, J66-J84, J90-J99, R04-R06, R84, R91
10	Liver cirrhosis	Liver cirrhosis	B18, F10, K70-K77, R16-R18, X45, Y15, Y90-91
11	Other non- communicable diseases	Sickle cell with crisis, Acute abdomen, Renal failure, Epilepsy, Congenital malformation, Other and unspecified, Other and unspecified NCD	D55-D63, D65-D83, D86, D89, E03-E07, E15-E35, E65-E68, E70-E90, F00-F09, F11-F52, F54-F99, G10-G37, G40-G41, G50-G80, G84-G99, H00-H06, H11-H59, H61-H62, H69, H72-H95, K00-K01, K03, K06-K14, K20-K31, K35-K38, K40-K60, K62-K64, K66, K78-K80, K82-K93, L05, L10-L99, M02-M54, M61-M85, M87-M99, N00-N08, N11-N29, N31-N33, N35-N40, N42-N48, N50-N59, N75-N86, N88-N99, Q00-Q99, R10-R15, R19-R23, R26-R27, R29-R49, R56, R63, R70-R74, R76-R77, R80-R82, R85-R87, R90, ZZ25
12	Neonatal conditions	Cause of death unknown, Prematurity, Birth asphyxia, Other and unspecified neonatal CoD	C76, D64, G40, O60, P00, P01, P02-P03, P05, P07, P10-P15, P21, P22, P24-P29, P50-P52, P61, P77, P80, P90-P92, R04, R06, Q00-Q99, W79, Z37
13	Road and transport injuries (RTI)	Road traffic accident, Other transport accident	V01-V99, Y85
14	Other injuries	Accid fall, Accid drowning and submersion, Accid expos to smoke fire & flame, Contact with venomous plant/animal, Accid poisoning & noxious subs, Assault, Exposure to force of nature, Other and unspecified external CoD	S00-S99, T00-T99, W00-W99, X00-X44, X46-X52, X55-X59, X85-X99, Y00-Y14, Y16-Y84, Y86-Y89, Y92-Y98, ZZ27
15	Ill-defined	NA	P96, R02, R07-R09, R25, R51-R54, R57-R58, R60-R62, R64-R69, R78-R79, R83, R89, R92-R94, R96, R98-R99
16	Suicide	Intentional self-harm	X60-X84
17	Maternal	Ectopic pregnancy, Abortion-related death, Pregnancy-induced hypertension, Obstetric haemorrhage, Obstructed labour, Pregnancy- related sepsis, Anaemia of pregnancy, Ruptured uterus, Other and unspecified maternal CoD, Not pregnant or recently delivered, Pregnancy ended within 6 weeks of death, Pregnant at death, Birth asphyxia, Fresh stillbirth, Macerated stillbirth	A34, F53, O00-O08, O10-O16, O20-O99

One may also argue for the use of more recent algorithm versions, such as InterVA-5, for assessments. Due to the fact that the VA data used were captured prior to the release of the WHO 2016 forms, the resultant binary files would have many missing symptoms. Furthermore, InterVA-5 was only recently released for public use, specifically in September of 2018. Although an enhanced algorithm may perform more effectively due to logic employed, the VA data is also very relevant for performance. Since the VA data used in this study conformed better with the 2014 forms, we ran experiments using algorithms that were designed from WHO 2014 VA forms or did not require a specific input for a fair comparison.

VA datasets also differ in COD composition counts; there are some CODs in the VA datasets which have large number of records, while other CODs have fewer records. The ratio of composition of these CODs is highly imbalanced which can make any algorithm more biased towards the CODs with higher ratio of records in the training set. This implies that the overall agreement would most likely remain low for the algorithms in such cases. COD balancing can be performed by duplicating the number of records for the minority CODs (CODs with the least amounts of records) or decreasing the number of records for the majority CODs (CODs with the greatest amounts of records) ^20^. However, these types of artificial balancing approaches do not always yield improvements in results.

A point for discussion relates to the distribution of CODs in training and test datasets. In machine learning, the composition of records of classes (e.g., CODs) are kept in the same proportion in the training and test set as in the original dataset when performing experiments ^20^. This allows for a fair evaluation of the algorithm, otherwise too many VA records in a test set of a COD and too few in the training set would only result in poor performance of the algorithm for that COD. In real life situations, when a machine learning application is in production, it is possible that we may not get all the variations in the training (historical) set and we may have more variations of a particular COD in the newly collected data. The common solution to this problem is to update the training data, and re-train the algorithm to reflect newer SCI variations as they are observed ^20^. Nonetheless, to understand the effect of different variations of CODs in training and test set, we performed another experiment by using Dirichlet distribution, which allowed us to vary the composition of records in the test set ^32^. We used Dirichlet distribution-based sampling that actually models variability in occurrences of classes (CODs) by applying resampling with replacement. We divided the dataset into 10 parts using 10-fold cross validation method ^20^ as in our experiments above. On each fold, we resampled the test set with replacement using Dirichlet distribution ^32^, resulting in different number of records for each type of COD. OAA-NBC, InterVA-4, Tariff, InSilicoVA and NBC were then evaluated on the resampled test set with different distribution of CODs. The results are shown in Table 5 for Matlab and MDS datasets. The overall performance of classification decreased as expected because the CODs with too few VA records in the actual training set were duplicated many times by the Dirichlet distribution in the new test set only. For example, if a record related to COD was not classified correctly by an algorithm and it was repeated many times in the test set then sensitivity would decrease on that COD. OAA-NBC and NBC still yielded better performance than all other algorithms. We showed results for these two datasets only as the other VA datasets had similar results of a dip in performance. An ideal training dataset would be a large repository of community VA deaths with enough variations in symptom patterns for each COD that are clinically verified; however, no such repository exists. The whole purpose of training on VA datasets is to be able to help classify CODs in situations where deaths occur unattended.

Finally, the performance of machine learning algorithms depend on the logic employed by the algorithm and the VA data, in terms of generating an adequate SCI for COD classification to discriminate different classes (CODs). To mitigate the effects of using one set of training data on all VA data, we trained algorithms on data derived from its origin dataset by using 10-fold cross validation method. By doing so, only SCIs generated from each separate VA data was considered when algorithms were classifying deaths per VA dataset. For the most part, the algorithms performed consistently, with OAA NBC performing better the majority of the time. Our results are reproducible; all of the scripts used and sample datasets are publicly available (see Experimental Setup section).

## Conclusion

In this study, we have enhanced the NBC algorithm using the one-against-all approach to assign CODs to records in multiple VA datasets from different settings. The results show that our approach has 6-8% better sensitivity and PCCC for individual-level COD classification than some of the current best performing computer-coded VA algorithms (i.e., Tariff, InterVA-4, NBC and InSilicoVA). Population-level agreements for OAA-NBC and NBC are found to be similar or higher than the other algorithms used in the experiments. Overall results show that OAA-NBC classification results are most like dual physician assignment based on VA data and clinical diagnostic COD assignments when compared against some of the leading algorithms by using cumulative sensitivity, PCCC and CSMF accuracy scores. The performance results are not due to chance as indicated by the Wilcoxon Signed Rank.

Thus, we conclude that using the one-against-all approach with NBC helps improve accuracy of COD classification. The one-against-all approach (and other ensemble methods of machine learning) can also be used with other VA algorithms instead of just Naïve Bayes. Although OAA-NBC generates the highest cumulative CSMF accuracy values, OAA-NBC still requires improvements to produce the most accurate COD classifications, especially for individual-level classification which is still below 80%. In the future, we plan to extend this work to include narratives present in the VA surveys for automated classification. Another endeavour would be to apply the one-against-all approach to the other algorithms to determine whether they can be improved further to classify community VA deaths more similarly to dual physician review. We also plan to explore different features selection techniques and prediction weighting methods (e.g., using CSMF distribution) for each individual NBC in OAA-NBC approach.

## Data availability

Some of the data used in the analysis has already been made available, specifically the PHMRC data which can be found at: http://ghdx.healthdata.org/record/population-health-metrics-research-consortium-gold-standard-verbal-autopsy-data-2005-2011.

The other datasets are included with the source code: https://github.com/sshahriyar/va (archived at https://doi.org/10.5281/zenodo.1489267 ^31^).

## Software availability

**Source code available from:** https://github.com/sshahriyar/va

**Archived source code at time of publication:** https://doi.org/10.5281/zenodo.1489267 ^31^.

**License:** MIT License.

## Supplementary material

**Supplementary file 1: Supplementary information for all datasets and algorithms.**
