# Supplementary material for: Automated verbal autopsy classification: using one-against-all ensemble method and Naïve Bayes classifier
Source: Gates Open Res. 2019 Jan 23;2:63. Originally published 2018 Nov 28. [Version 2] doi: 10.12688/gatesopenres.12891.2 (PMC6480413; doi:10.12688/gatesopenres.12891.2)
Supplement: Supplementary file 1 [file gatesopenres-2-14008-s0000.tgz › 7e793fba-a720-4d60-a281-55b2653f209d_Supplementary_file_1.docx]

# Supplementary file 1

In this section, we provide supplementary information for all datasets and algorithms. Results of rank 2 to rank 5 are the sum of all the previous results till that rank. Note that the values are not represented as percentages (maximum value will be 1.00 instead of 100.0).

Table A.1: Performance of algorithms on MDS dataset

|  |  | Rank1 | Rank2 | Rank3 | Rank4 | Rank5 |
| --- | --- | --- | --- | --- | --- | --- |
| Sensitivity | Tariff | 0.315 | 0.477 | 0.580 | 0.657 | 0.714 |
|  | InterVA4 | 0.488 | 0.633 | 0.725 | 0.784 | 0.827 |
|  | InSilicoVA | 0.456 | 0.630 | 0.735 | 0.806 | 0.859 |
|  | NBC | 0.560 | 0.718 | 0.808 | 0.866 | 0.901 |
|  | **OAA-NBC** | **0.611** | **0.743** | **0.870** | **0.917** | **0.943** |
| PCCC | Tariff | 0.266 | 0.396 | 0.475 | 0.532 | 0.571 |
|  | InterVA4 | 0.452 | 0.576 | 0.656 | 0.706 | 0.741 |
|  | InSilicoVA | 0.417 | 0.572 | 0.668 | 0.735 | 0.788 |
|  | NBC | 0.529 | 0.676 | 0.763 | 0.821 | 0.856 |
|  | **OAA-NBC** | **0.585** | **0.744** | **0.837** | **0.886** | **0.915** |
| CSMF Acc. | Tariff | 0.575 | 0.688 | 0.750 | 0.780 | 0.808 |
|  | InterVA4 | 0.710 | 0.792 | 0.839 | 0.866 | 0.881 |
|  | InSilicoVA | 0.707 | 0.829 | 0.875 | 0.908 | 0.931 |
|  | NBC | 0.867 | 0.912 | 0.933 | 0.942 | 0.948 |
|  | **OAA-NBC** | **0.890** | **0.956** | **0.967** | **0.980** | **0.982** |

Table A.2: Performance of algorithms on Matlab dataset.

|  |  | Rank1 | Rank2 | Rank3 | Rank4 | Rank5 |
| --- | --- | --- | --- | --- | --- | --- |
| Sensitivity | Tariff | 0.407 | 0.576 | 0.657 | 0.716 | 0.753 |
|  | InterVA4 | 0.348 | 0.529 | 0.649 | 0.729 | 0.793 |
|  | InSilicoVA | 0.356 | 0.515 | 0.655 | 0.741 | 0.808 |
|  | NBC | 0.507 | 0.670 | 0.769 | 0.838 | 0.872 |
|  | **OAA-NBC** | **0.579** | **0.735** | **0.813** | **0.870** | **0.912** |
| PCCC | Tariff | 0.365 | 0.510 | 0.571 | 0.612 | 0.630 |
|  | InterVA4 | 0.301 | 0.457 | 0.561 | 0.630 | 0.689 |
|  | InSilicoVA | 0.310 | 0.440 | 0.568 | 0.648 | 0.712 |
|  | NBC | 0.472 | 0.619 | 0.711 | 0.779 | 0.809 |
|  | **OAA-NBC** | **0.549** | **0.697** | **0.776** | **0.824** | **0.867** |
| CSMF Acc. | Tariff | 0.615 | 0.741 | 0.770 | 0.795 | 0.819 |
|  | InterVA4 | 0.612 | 0.726 | 0.776 | 0.800 | 0.845 |
|  | InSilicoVA | 0.618 | 0.729 | 0.814 | 0.857 | 0.900 |
|  | NBC | 0.839 | 0.900 | 0.919 | 0.923 | 0.935 |
|  | **OAA-NBC** | **0.863** | **0.923** | **0.929** | **0.938** | **0.950** |

Table A.3: Performance of algorithms on Agincourt dataset.

|  |  | Rank1 | Rank2 | Rank3 | Rank4 | Rank5 |
| --- | --- | --- | --- | --- | --- | --- |
| Sensitivity | Tariff | 0.275 | 0.462 | 0.570 | 0.661 | 0.721 |
|  | InterVA4 | 0.463 | 0.632 | 0.689 | 0.740 | 0.788 |
|  | InSilicoVA | 0.358 | 0.558 | 0.661 | 0.743 | 0.803 |
|  | NBC | 0.482 | 0.676 | 0.763 | 0.828 | 0.874 |
|  | **OAA-NBC** | **0.555** | **0.743** | **0.834** | **0.895** | **0.931** |
| PCCC | Tariff | 0.227 | 0.385 | 0.471 | 0.548 | 0.595 |
|  | InterVA4 | 0.427 | 0.580 | 0.617 | 0.654 | 0.692 |
|  | InSilicoVA | 0.316 | 0.495 | 0.584 | 0.658 | 0.714 |
|  | NBC | 0.447 | 0.630 | 0.709 | 0.771 | 0.816 |
|  | **OAA-NBC** | **0.525** | **0.706** | **0.796** | **0.859** | **0.901** |
| CSMF Acc. | Tariff | 0.594 | 0.709 | 0.755 | 0.800 | 0.816 |
|  | InterVA4 | 0.705 | 0.746 | 0.786 | 0.831 | 0.874 |
|  | InSilicoVA | 0.665 | 0.799 | 0.827 | 0.874 | 0.887 |
|  | NBC | 0.840 | 0.920 | 0.907 | 0.937 | 0.941 |
|  | **OAA-NBC** | **0.881** | **0.925** | **0.948** | **0.964** | **0.972** |

Table A.4: Performance of algorithms on PHMRC adult-global dataset.

|  |  | Rank1 | Rank2 | Rank3 | Rank4 | Rank5 |
| --- | --- | --- | --- | --- | --- | --- |
| Sensitivity | Tariff | 0.359 | 0.513 | 0.613 | 0.679 | 0.747 |
|  | InterVA4 | 0.363 | 0.560 | 0.680 | 0.755 | 0.822 |
|  | InSilicoVA | 0.350 | 0.513 | 0.627 | 0.720 | 0.795 |
|  | NBC | 0.477 | 0.654 | 0.764 | 0.837 | 0.881 |
|  | **OAA-NBC** | **0.531** | **0.702** | **0.802** | **0.868** | **0.910** |
| PCCC | Tariff | 0.305 | 0.424 | 0.497 | 0.537 | 0.590 |
|  | InterVA4 | 0.310 | 0.480 | 0.584 | 0.647 | 0.710 |
|  | InSilicoVA | 0.296 | 0.425 | 0.515 | 0.596 | 0.667 |
|  | NBC | 0.433 | 0.591 | 0.693 | 0.765 | 0.807 |
|  | **OAA-NBC** | **0.491** | **0.648** | **0.743** | **0.810** | **0.854** |
| CSMF Acc. | Tariff | 0.660 | 0.756 | 0.792 | 0.813 | 0.857 |
|  | InterVA4 | 0.658 | 0.808 | 0.848 | 0.872 | 0.893 |
|  | InSilicoVA | 0.670 | 0.770 | 0.820 | 0.867 | 0.902 |
|  | NBC | 0.859 | 0.933 | 0.943 | 0.957 | 0.960 |
|  | **OAA-NBC** | **0.903** | **0.948** | **0.959** | **0.963** | **0.968** |

Table A.5: Performance of algorithms on PHMRC children-global dataset.

|  |  | Rank1 | Rank2 | Rank3 | Rank4 | Rank5 |
| --- | --- | --- | --- | --- | --- | --- |
| Sensitivity | Tariff | 0.370 | 0.553 | 0.686 | 0.766 | 0.837 |
|  | InterVA4 | 0.451 | 0.640 | 0.760 | 0.847 | 0.918 |
|  | InSilicoVA | 0.433 | 0.588 | 0.725 | 0.816 | 0.896 |
|  | NBC | 0.515 | 0.684 | 0.799 | 0.877 | 0.931 |
|  | **OAA-NBC** | **0.546** | **0.716** | **0.820** | **0.891** | **0.934** |
| PCCC | Tariff | 0.291 | 0.425 | 0.529 | 0.579 | 0.633 |
|  | InterVA4 | 0.382 | 0.537 | 0.640 | 0.725 | 0.817 |
|  | InSilicoVA | 0.362 | 0.471 | 0.587 | 0.669 | 0.767 |
|  | NBC | 0.454 | 0.594 | 0.699 | 0.779 | 0.846 |
|  | **OAA-NBC** | **0.490** | **0.635** | **0.731** | **0.805** | **0.851** |
| CSMF Acc. | Tariff | 0.737 | 0.830 | 0.855 | 0.877 | 0.905 |
|  | InterVA4 | 0.778 | 0.878 | 0.903 | 0.925 | 0.953 |
|  | InSilicoVA | 0.807 | 0.848 | 0.908 | 0.931 | 0.951 |
|  | NBC | 0.911 | 0.921 | 0.937 | 0.953 | 0.968 |
|  | **OAA-NBC** | **0.901** | **0.915** | **0.943** | **0.959** | **0.968** |

Table A.6: Performance of algorithms on PHMRC adult-Indian dataset.

|  |  | Rank1 | Rank2 | Rank3 | Rank4 | Rank5 |
| --- | --- | --- | --- | --- | --- | --- |
| Sensitivity | Tariff | 0.440 | 0.583 | 0.669 | 0.739 | 0.794 |
|  | InterVA4 | 0.411 | 0.588 | 0.677 | 0.806 | 0.846 |
|  | InSilicoVA | 0.503 | 0.663 | 0.763 | 0.822 | 0.873 |
|  | NBC | 0.548 | 0.690 | 0.757 | 0.815 | 0.861 |
|  | **OAA-NBC** | **0.601** | **0.757** | **0.835** | **0.886** | **0.931** |
| PCCC | Tariff | 0.393 | 0.507 | 0.570 | 0.624 | 0.665 |
|  | InterVA4 | 0.362 | 0.513 | 0.580 | 0.720 | 0.751 |
|  | InSilicoVA | 0.462 | 0.602 | 0.692 | 0.743 | 0.794 |
|  | NBC | 0.509 | 0.633 | 0.684 | 0.734 | 0.774 |
|  | **OAA-NBC** | **0.567** | **0.713** | **0.786** | **0.836** | **0.889** |
| CSMF Acc. | Tariff | 0.696 | 0.752 | 0.792 | 0.830 | 0.859 |
|  | InterVA4 | 0.697 | 0.811 | 0.819 | 0.868 | 0.901 |
|  | InSilicoVA | 0.836 | 0.895 | 0.905 | 0.929 | 0.929 |
|  | NBC | 0.891 | 0.895 | 0.882 | 0.899 | 0.907 |
|  | **OAA-NBC** | **0.892** | **0.932** | **0.929** | **0.939** | **0.957** |

Table A.7: Performance of algorithms on PHMRC children-Indian dataset.

|  |  | Rank1 | Rank2 | Rank3 | Rank4 | Rank5 |
| --- | --- | --- | --- | --- | --- | --- |
| Sensitivity | Tariff | 0.395 | 0.559 | 0.666 | 0.745 | 0.863 |
|  | InterVA4 | 0.512 | 0.715 | 0.809 | 0.887 | 0.930 |
|  | InSilicoVA | 0.494 | 0.671 | 0.811 | 0.881 | 0.924 |
|  | NBC | 0.586 | 0.734 | 0.833 | 0.886 | 0.924 |
|  | **OAA-NBC** | **0.630** | **0.782** | **0.876** | **0.917** | **0.947** |
| PCCC | Tariff | 0.320 | 0.433 | 0.499 | 0.542 | 0.693 |
|  | InterVA4 | 0.451 | 0.634 | 0.713 | 0.796 | 0.843 |
|  | InSilicoVA | 0.431 | 0.578 | 0.716 | 0.787 | 0.829 |
|  | NBC | 0.534 | 0.658 | 0.750 | 0.794 | 0.829 |
|  | **OAA-NBC** | **0.581** | **0.720** | **0.815** | **0.851** | **0.881** |
| CSMF Acc. | Tariff | 0.715 | 0.772 | 0.828 | 0.842 | 0.931 |
|  | InterVA4 | 0.780 | 0.864 | 0.891 | 0.933 | 0.953 |
|  | InSilicoVA | 0.800 | 0.877 | 0.905 | 0.930 | 0.950 |
|  | NBC | 0.834 | 0.895 | 0.912 | 0.912 | 0.934 |
|  | **OAA-NBC** | **0.834** | **0.895** | **0.931** | **0.950** | **0.967** |

Table A.8: Performance of algorithms on Matlab Dataset using Dirichlet distribution.

|  |  | Rank1 | Rank2 | Rank3 | Rank4 | Rank5 |
| --- | --- | --- | --- | --- | --- | --- |
| Sensitivity | Tariff | 0.452 | 0.599 | 0.685 | 0.760 | 0.790 |
|  | InterVA4 | 0.334 | 0.467 | 0.564 | 0.663 | 0.715 |
|  | InSilicoVA | 0.377 | 0.538 | 0.686 | 0.759 | 0.814 |
|  | NBC | 0.387 | 0.534 | 0.631 | 0.692 | 0.737 |
|  | **OAA-NBC** | **0.456** | **0.631** | **0.728** | **0.792** | **0.862** |
| PCCC | Tariff | 0.339 | 0.454 | 0.573 | 0.607 | 0.650 |
|  | InterVA4 | 0.286 | 0.385 | 0.456 | 0.541 | 0.572 |
|  | InSilicoVA | 0.333 | 0.467 | 0.607 | 0.672 | 0.722 |
|  | NBC | 0.343 | 0.463 | 0.539 | 0.580 | 0.606 |
|  | **OAA-NBC** | **0.417** | **0.574** | **0.660** | **0.717** | **0.793** |
| CSMF Acc. | Tariff | 0.546 | 0.659 | 0.738 | 0.790 | 0.808 |
|  | InterVA4 | 0.516 | 0.619 | 0.645 | 0.717 | 0.751 |
|  | InSilicoVA | 0.594 | 0.687 | 0.783 | 0.816 | 0.858 |
|  | NBC | 0.576 | 0.673 | 0.716 | 0.767 | 0.767 |
|  | **OAA-NBC** | **0.604** | **0.754** | **0.797** | **0.843** | **0.883** |

Table A.9: Performance of algorithms on MDS Dataset using Dirichlet distribution.

|  |  | Rank1 | Rank2 | Rank3 | Rank4 | Rank5 |
| --- | --- | --- | --- | --- | --- | --- |
| Sensitivity | Tariff | 0.290 | 0.411 | 0.523 | 0.594 | 0.647 |
|  | InterVA4 | 0.336 | 0.451 | 0.526 | 0.592 | 0.639 |
|  | InSilicoVA | 0.381 | 0.538 | 0.645 | 0.709 | 0.759 |
|  | NBC | 0.417 | 0.543 | 0.639 | 0.708 | 0.747 |
|  | **OAA-NBC** | **0.410** | **0.535** | **0.618** | **0.703** | **0.750** |
| PCCC | Tariff | 0.239 | 0.321 | 0.404 | 0.447 | 0.470 |
|  | InterVA4 | 0.289 | 0.367 | 0.407 | 0.443 | 0.459 |
|  | InSilicoVA | 0.336 | 0.466 | 0.555 | 0.602 | 0.636 |
|  | NBC | 0.375 | 0.472 | 0.547 | 0.600 | 0.619 |
|  | **OAA-NBC** | **0.370** | **0.474** | **0.533** | **0.590** | **0.625** |
| CSMF Acc. | Tariff | 0.537 | 0.604 | 0.678 | 0.717 | 0.746 |
|  | InterVA4 | 0.494 | 0.552 | 0.621 | 0.681 | 0.707 |
|  | InSilicoVA | 0.572 | 0.697 | 0.758 | 0.783 | 0.805 |
|  | NBC | 0.604 | 0.668 | 0.737 | 0.776 | 0.796 |
|  | **OAA-NBC** | **0.598** | **0.656** | **0.730** | **0.756** | **0.792** |
