# Supplementary material for: Automated verbal autopsy classification: using one-against-all ensemble method and Naïve Bayes classifier
Source: Gates Open Res. 2019 Jan 23;2:63. Originally published 2018 Nov 28. [Version 2] doi: 10.12688/gatesopenres.12891.2 (PMC6480413; doi:10.12688/gatesopenres.12891.2)
Supplement: Peer review history

# Peer review: Reviewer response for version 2

**DOI:** 10.21956/gatesopenres.14008.r26882

**Published:** 2019-02-08

**Authors:** Aaron S. Karat, Clara Calvert

**Affiliations:** 1 Department of Clinical Research, London School of Hygiene & Tropical Medicine, London, UK; 2 Department of Population Health, London School of Hygiene & Tropical Medicine, London, UK

We thank the authors for their detailed response and for the changes made to the manuscript. We are happy to approve - congratulations on this important piece of work!

We have read this submission. We believe that we have an appropriate level of expertise to confirm that it is of an acceptable scientific standard.

# Peer review: Reviewer response for version 1

**DOI:** 10.21956/gatesopenres.13987.r26787

**Published:** 2019-01-03

**Authors:** Ying Lu

**Affiliations:** 1 Department of Applied Statistics, Social Sciences and Humanities, Steinhardt School of Education, Culture and Human Development, New York University, New York, NY, USA

First I would like to congratulate the authors for developing an effective solution to the verbal autopsy classification problem. The results look very convincing, and the rationale of the methods seems to be reasonable. The source code is open-access. 

 I have several questions / suggestions for the authors:

1. How well does the one-against-all method perform when the number of disease categories increases? Will the uncertainty go up significantly?
2. Since different NBC is fit for each COD, the probability of particular cause predicted for each death will be different. When the final cause is determined, it seems that these individual probabilities should be weighted rather than just simply taking the max of all. The weights can be chosen to be the values that will optimize the overall cause specific mortality rate distribution (CSMFs). 
3. For each NBC, it seems that some feature selections can be done to improve the accuracy of these individual predictions. 

I have read this submission. I believe that I have an appropriate level of expertise to confirm that it is of an acceptable scientific standard.

# Response

**Published:** 2019-01-10

**Authors:** Syed Shariyar Murtaza

**Affiliations:** Ryerson University, Canada

Thank you for reviewing this article. Please find below replies to your questions.

**Q1.** How well does the one-against-all method perform when the number of disease categories increases? Will the uncertainty go up significantly?

**REPLY 1:** We are not sure if the reviewers mean CODs by disease categories or symptoms (features). We will answer from both perspectives.

- Number of CODs increases:

It will depend on the dataset. If we add a new COD with few (e.g., 10) records in the dataset, then the accuracy of classification will decrease slightly (e.g., approx. 1-2%). This is because a small ratio of records is not helpful in classification when some other CODs have very high ratio of records (e.g., few thousand records). Also, if the newer COD has most of the symptoms similar to another COD, then there are no sufficient discriminating factors between records of CODs. The accuracy of classification of this new category would remain low in this case too. If number of records are sufficient in ratio (e.g., at least 50-100) for newer category and there is sufficient discrimination in terms of symptoms then the machine learning approaches, like one-against-all method with Naïve Bayes algorithm, will be able to classify records with good accuracy. In the case of current VA datasets, they accuracy of classification can be improved by increasing the ratio of records for categories of diseases that have a very small ratio of records compared to others and also by introducing better discriminating symptoms. The newer symptoms can be synthetic too by using other approaches in machine learning (see Answer 3).

- Number of Symptoms (Features) Increases:

If the number of symptoms increase and they increase the discriminating power between CODs then the accuracy will improve; otherwise, the increase in symptoms will not affect accuracy or will decrease the accuracy.

**Q2.** Since different NBC is fit for each COD, the probability of particular ......... overall cause specific mortality rate distribution (CSMFs).

**REPLY 2:** Each NBC generates a probability of COD and final list of predicted CODs from all NBCs is generated by sorting them in descending order by their probabilities. However, not all NBCs predict a COD with a probability, some NBCs also predict the cause “Others”—recall that each NBC has two causes to predict: COD and “Others”. When “Others” cause is predicted then it means that NBC is predicting that the COD (that it knows) is not the real cause, and we can simply ignore “Others” prediction. In this way, for 15 NBCs there are different numbers of predicted CODs in the final list depending on the VA record.

It is a good suggestion to weight the predictions of CODs and then sort the predicted CODs by their final weighted probabilities. CSMF distribution is highly imbalanced for CODs in the VA datasets. So assigning weights proportional to the CMSF distribution would increase the chances of prediction of CODs in majority but they are already predicted accurately because of their large number of records. This could eventually decrease the accuracy. However, in our view a better way would be use the weights inversely proportional to CSMF distributions because that would give a better chance to those CODs which have fewer records and  which are not correctly predicted by individual NBCs. This is a very good direction of research, we would like to explore this further in our future work and added to the future work section of our paper.

**Q3.** For each NBC, it seems that some feature selections can be done to improve the accuracy of these individual predictions.

**REPLY 3:** This is correct, better discriminating symptoms (features) can improve the accuracy of prediction for each COD (i.e., each NBC). Feature selection can be done subjectively by using expert judgements or by using feature selection algorithms in machine learning.  Accuracy could also be improved by introducing additional features, those features could be synthetic too; e.g., a feature X can be transformed into a new feature X+c where c is a constant, by taking its power such as X1/2, and by using similar such techniques. This could generate a new feature space that could help in better classifying the CODs. There are many feature selection methods and feature transformation methods. This will require another set of exploratory experiments to determine which one can actually improve accuracy of classification of CODs. This is a good direction of future work and we have added to the future work section of our paper.

# Peer review: Reviewer response for version 1

**DOI:** 10.21956/gatesopenres.13987.r26788

**Published:** 2018-12-20

**Authors:** Aaron S. Karat, Clara Calvert

**Affiliations:** 1 Department of Clinical Research, London School of Hygiene & Tropical Medicine, London, UK; 2 Department of Population Health, London School of Hygiene & Tropical Medicine, London, UK

Thank you for the opportunity to review this article: it describes the development of a new method in an important area of global health and for the most part is well written and organised. Overall, the authors make a coherent argument, though we have a few suggestions on how certain aspects could be clarified or improved.

**Introduction**

1. This provides a good overview of the current state of automated VA classification (though describing King-Lu as a ‘current leading’ method seems a bit of a stretch). The justification for the development of this method could be fleshed out a little more, perhaps explaining (for those unfamiliar with how VA data feed into policy) why it is important for these methods to be more accurate. To this end, the authors may want to consider citing the 2014 systematic review by Leitao et al. comparing PCVA with CCVA in LMIC and mentioning - even briefly - the large project underway to incorporate VA into CRVS systems ( https://crvsgateway.info/A-stepwise-process~503).
2. The NBC is the model chosen for testing the one-against-all approach – it would be good to include a couple of sentences justifying this choice based on previous literature before the final paragraph in the introduction. 
3. In general, we found the use of the term ‘CoD diagnosis’ (used in the introduction and elsewhere in the manuscript) a little confusing. We would suggest using ‘assignment’ consistently throughout, to differentiate from ‘diagnoses’ made by clinicians during life.

**Methods**

1. Though it is made reasonably clear in the text that the MDS, Agincourt, and Matlab CoD are based on physician review of VA data compared with physician review of clinical data for PHMRC CoD, we think that this distinction could (and should) be made more clearly and repeatedly throughout the manuscript, including in Tables 1, 2, and 4. As the authors are no doubt aware, the use of PCVA CoD as a gold standard is not ideal, constituting, to some extent, a ‘circular’ comparison, as both methods ultimately rely on the quality of the VA data. We feel that the authors could make greater efforts to make clear (to the non-expert reader) this key difference between the different datasets. (Note the justification for the use of PCVA CoD as gold standard [page 6, under ‘assessment methods’] does not really address this issue – a high level of agreement between physicians reading the same VA data does not have any bearing on the objective ‘truth’ of their assignments.)
2. A minor point: the use of ‘historical’ and ‘new’ VA surveys in Figure 1 is potentially misleading, as it suggests that new data were collected and used to test the algorithm/s. Would ‘train’ and ‘test’ be more appropriate?
3. Figure 2 is a helpful representation of the OAA approach. It may be useful to combine figures 1 and 2, showing in one place the workings of the method and how it fits into the process and, perhaps, showing in more detail how outputs from the multiple models are ‘re-assembled’ to give one list of causes and probabilities that can then be interpreted or compared with the outputs from other methods.
4. The authors provide a detailed description of the methods used to compare the CoD assigned by different methods, citing the guidance from Murray et al. in 2011 (reference 26). However, they do not report the chance-corrected CSMF accuracy (as described by Flaxman et al. (2015) ^1^) – could the reasons for this be mentioned? 
5. Referring to CSMF accuracy as “agreement” is potentially confusing – we would suggest using the full term or “CSMFa” throughout.
6. The description of the calculation of cumulative sensitivity is a little confusing. Does the ‘15% more correct at rank 2’ include only those which are also correct at rank 1? i.e., if methods corresponded at rank 2 but not at rank 1, would they be included in the cumulative estimate? This is not a method previously described in the VA literature and is fairly central to the interpretation of the results presented, so more detail is necessary. It would also be helpful to provide some justification for the choice of reporting cumulative sensitivity to rank 5.
7. A minor point: the description of computing the ‘average’ sensitivity, PCCC, and agreement (page 7, column 2, end of paragraph 1) is a little vague – please consider using ‘mean’ or another appropriate technical term.

 

**Results**

1. It is not clear from the text in paragraph 1 of the results that agreements for ‘rank 5’ are cumulative from ranks 1 to 5. Stating that the most likely *and* the fifth most likely were used implies that ranks 2–4 were excluded. Similarly, in Table 3, although ‘cumulative sensitivity’ is mentioned, the authors may want to consider changing the column headers from ‘Rank 5’ to ‘Ranks 1–5’, to signpost more clearly what the numbers represent.
2. The decision to display only estimates of sensitivity, without any estimates of PCCC, is defended in the text; however, for full transparency and to allow for comparison with other similar studies, none of which (to our knowledge) report on cumulative sensitivity, the authors should consider including these results in a supplementary table/appendix. It would also be helpful to provide numeric values for the estimates of CSMF accuracy presented in Figure 3.
3. It is not clear what exactly was being tested when the authors write “we conducted the Wilcoxon signed rank test on 35 observations of agreements for the five algorithms” (page 9, second paragraph under “Ranked sensitivity comparison”). We assume that the PCCC values were being tested, but where does the 35 come from? It would be helpful to have more detail on this in the methods section.
4. If possible, please provide the exact p-values (or at least a range of values) from applying the Wilcoxon signed rank test to the population-level agreements; alternatively, these could be provided in a supplementary table. It would also be helpful to clarify whether there was any evidence that sensitivity was statistically different between OAA-NBC and NBC and include a p-value for this.
5. A minor point: when discussing the Wilcoxon signed rank statistical test, it is written that “we also included rank two and rank three values” – what about rank 4 values? Why would this be left out?

 

**Discussion**

1. A number of results are described in the discussion section (results of testing pre-trained models and Dirichlet distributions-based samples [Table 5]). Might these more appropriately be moved to the results, with the corresponding methods described and tables included as appendices as needed?
2. In Table 6, it is not clear why ‘HIV/AIDS-related deaths’ (ICD-10 codes B20–B24) are included under ‘other and unspecified infections’. Is this an error? This does not correspond with the cause-specific sensitivities shown in Table 4 - please could the authors clarify?
3. It would be pertinent to acknowledge, again, the difference between the ‘reference standards’ used for comparison and to discuss (even briefly) the potential implications of using CoD derived from VA data as reference. Greater clarity in describing the two reference standards would also be useful; for example, in the first paragraph under ‘Conclusions’, describing “dual physician *assignment based on* *VA data* and clinical diagnostic COD…” would more clearly make the distinction.
4. It would be useful to include a paragraph comparing the results of this exercise to previous validation exercises done on the algorithms; did the authors find similar results to, for example, James et al. (Pop Health Met 2011)? If not, what are the differences in the exercises undertaken?
5. The reporting of cumulative sensitivity as the main measure of agreement is an unusual aspect of this study; acknowledgment of this as a potential limitation would help provide context for comparisons with other similar studies.

 

**Minor points**

1. In the abstract, the authors write: “The results demonstrate that our approach improves the classification from 6% to 8%”, which could be interpreted as suggesting that sensitivity was only 6% in the other algorithms. Perhaps this could be re-phrased along the lines of “The results demonstrate that our approach improves the classification by between 6% and 8% compared with the other algorithms”.
2. The Matlab and Agincourt datasets are referred to as DHS – it would be better to refer to them as Health and Demographic Surveillance Sites (HDSS) to prevent confusion between these and the Demographic and Health Surveys.
3. The article switches from past to present tense a number of times; for example, the first paragraph of methods is (mostly) in present tense, but most of the rest of the methods is in past tense. For consistency and to improve readability, we would suggest re-writing these passages in past tense.
4. Probable typo: methods, paragraph 2, line 15 – “datasets” should be “dataset”?
5. Per normal conventions, please consider adding legends to tables and figures spelling out any acronyms used

We have read this submission. We believe that we have an appropriate level of expertise to confirm that it is of an acceptable scientific standard, however we have significant reservations, as outlined above.

## References

[1] FlaxmanADSerinaPTHernandezBMurrayCJRileyILopezAD: Measuring causes of death in populations: a new metric that corrects cause-specific mortality fractions for chance. *Popul Health Metr*.2015;13: 10.1186/s12963-015-0061-1 28 10.1186/s12963-015-0061-1 26464564PMC4603634

# Response

**Published:** 2019-01-10

**Authors:** Syed Shariyar Murtaza

**Affiliations:** Ryerson University, Canada

Thank you for reviewing this article. Please find below replies to your questions. We have also submitted a modified version with your recommendations.

**Introduction**

Q1. This provides a good overview of the current state of automated VA classification (though describing King-Lu as a ‘current leading’ method seems a bit of a stretch). ...................orate VA into CRVS systems ( https://crvsgateway.info/A-stepwise-process~503).

REPLY 1: We have made the modification in the Introduction Section.

Q2. The NBC is the model chosen for testing the one-against-all approach ........... introduction. 

REPLY 2: In our earlier version, we have discussed the justification in Methods section. We have also added a similar statement in the last paragraph of Introduction as suggested by reviewers.

Q3: In general, we found the use of the term ‘CoD diagnosis’....................made by clinicians during life.

REPLY 3: We have changed diagnosis to assignment to avoid confusion as suggested.

**Methods**

Q4. Though it is made reasonably clear in the text that the MDS, Agincourt, and Matlab CoD are based ....................... ‘truth’ of their assignments.)

REPLY 4: Table 4 already had a footnote; however, based on your suggestion we clarified it further and we added footnotes for Table 1 and Table 2.

Q5. A minor point: the use of ‘historical’ and ‘new’ VA surveys in ..............  ‘test’ be more appropriate?

REPLY 5: We have modified the figure with the inclusion of the terms suggested.

Q6. Figure 2 is a helpful representation of the OAA approach............... or compared with the outputs from other methods.

REPLY 6: This is a good suggestion but we would like to keep the figures separate as it would clutter one figure, and the abstraction makes it easier to understand. However, we made some changes in Figure 1 which captures your suggestion. The output of each model is assembled in a simple manner: COD predictions/assignments from all models are simply sorted by their probability of prediction/assignments.

Q7. The authors provide a detailed description of the methods ................................... be mentioned? 

REPLY 7:  CSMF accuracy is the most widely used measure in the VA assessment studies, and this is the primary reason for choosing it in our study too. Chance-corrected CSMF (CCCSMF) accuracy could have been used in our study but it would not have made any difference in the value of overall results other than reducing the CSMF accuracy values for each algorithm/method. This can be understood from this equation presented by Flaxman et al. for chance correcting previous results: CCCSMF= (CSMF- mean random allocation / 1 – mean random allocation). The mean random allocation values in this equation for a dataset are measured by performing random predictions using Dirichlet distribution many times and taking their mean. This would be a constant number for a dataset, and it would only end up reducing a CSMF accuracy value by a constant rate only.

Furthermore, we have shown results separately using Dirichlet distribution for different datasets and methods. We have also shown results on individual causes alongside individual sensitivity measures. All these different perspectives mitigate the doubts of incorrect reported performances of methods in our study. 

On another note, the use of Dirichlet distribution method only duplicates or reduces VA records in a training or test dataset, which actually only result in reduce performance of methods. An appropriate approach would be to have a training set with all variations of a cause of deaths that are expected to be observed in the field.

Q8. Referring to CSMF accuracy as “agreement” is potentially confusing – we would suggest using the full term or “CSMFa” throughout.

REPLY 8: We have changed agreement to CSMF accuracy throughout the entire paper as suggested.

Q9. The description of the calculation of cumulative sensitivity is a little confusing. ............  provide some justification for the choice of reporting cumulative sensitivity to rank 5.

REPLY 9: Reporting cumulative results is a popular approach used in applied machine learning and software engineering literature (see for example [1][2][3]). Random probability of prediction of causes of a problem is 1/N, where N is the number of causes. When data is not big, not separable, and has many causes, first rank prediction from any algorithm would not reach close to 100% mark.  It is then useful to know how an algorithm would fare on top few predictions of causes (e.g., top 3 ranks, top 5 ranks, etc.) because an accuracy of 90% on top 4 causes implies that there is a 25% (1/4) probability of 90% accurate sensitivity (predictions). This is better than reviewing N causes (15 approximately in our datasets) which has a probability of 6.6% success.

Yes, if an algorithm has 15% sensitivity at rank 1 and 20 % sensitivity at rank 2 then cumulative sensitivity would be 35% at rank 2. Sensitivity at rank N is the sum of sensitivity values from rank 1 to rank N. Consider a method A has sensitivity values for top two ranks 30% & 20%, and a method B has sensitivity values 20% & 30% for top 2 ranks. The cumulative sensitivity values at rank 2 for both methods A and B would be 50%.  However, this was not the case in our experiments. OAA-NBC consistently yielded better results at all ranks (from 1 to 5 and afterwards). The reason for choosing top 5 ranked predictions is subjective and it could have been top 4 or top 3 too.

The concept of cumulative reporting is straightforward, it does not affect traditional method of reporting results (which is only about first rank), and only adds additional information to the existing way of reporting. This should not be a source of concern for evaluation. We have modified text in the last paragraph of Assessment Methods section in Methods section to make the explanation clearer.

[1] S. S. Murtaza, N. H. Madhavji, M. Gittens and A. Hamou-Lhadj, "Identifying Recurring Faulty Functions in Field Traces of a Large Industrial Software System," in *IEEE Transactions on Reliability*, vol. 64, no. 1, pages 269-283,  2015.

[2] W. Wong,  V. Debroy, R. Golden, X. Xiaofeng, B. Thuraisingham, Effective software fault localization using  an RBF neural network,  IEEE Trans. Reliab, Issue 61, Vol 1, pages 149–169,  2012.

[3] S. S. Murtaza, A. Hamou-Lhadj, N. H. Madhavji, M. Gittens, An empirical study on the use of mutant traces for diagnosis of faults in deployed systems, Journal of Systems and Software, Volume 90, pages 29-44, 2014. 

Q10. A minor point: the description of computing the ‘average’ sensitivity, PCCC, and agreement (page 7, column 2, end of paragraph 1) is a little vague – please consider using ‘mean’ or another appropriate technical term.

REPLY 10: We have made the modification.

**Results**

Q11. It is not clear from the text in paragraph 1 of the results that agreements..........to signpost more clearly what the numbers represent.

REPLY 11: We have made the modifications everywhere in the text to further articulate that fifth rank represents the cumulative value from rank 1 to rank 5 as per your suggestion.

Q12. The decision to display only estimates of sensitivity, ................. estimates of CSMF accuracy presented in Figure 3.

REPLY 12: The reason for removing results (rank values, PCCC values, etc.) is to avoid cluttering of text with lots of tables and increase readability. We have added an appendix in the paper which reports all the results including sensitivity, PCC, CSMF accuracy and values at different ranks.

Q13. It is not clear what exactly was being tested when the ...........It would be helpful to have more detail on this in the methods section.

REPLY 13: 35 observations refer to 5 ranked (rank 1 to rank 5) prediction values across the seven VA datasets. So for each algorithm we have 35 observations of sensitivity values, PCC and CSMF values. All the data is now present in Appendix A. The word agreement has been removed and replaced with CSMF accuracy.

Q14. If possible, please provide the exact p-values (or at least a range of values) from applying the Wilcoxon signed rank test.......... OAA-NBC and NBC and include a p-value for this.

REPLY 14: We already provided the evidence of p values between OAA-NBC and NBC. Below is a sample from the text of Results section:

“We also performed a Wilcoxon signed rank statistical test on the reported sensitivity in Table 3, generated from the five…………….. the Wilcoxon signed ranked test yielded Z-score=5.194 and two tailed p-value=2.47 x 10 ^-7^ between OAA-NBC and NBC.”

For Wilcoxon test on CSMF accuracy we found  the Z-score=4.248 and p-value =2.15 x 10 ^-5^ between OAA-NBC and NBC. Exactly same values were also obtained for test of OAA-NBC and other algorithms in a pairwise manner.

The p values are extremely small in all the comparisons of OAA-NBC against other algorithms for both sensitivity and CSMF accuracy. Since the values are the same (for CSMF and for sensitivity; see Results Section), it is not worth showing these many similar p values, especially now all data is present in Appendix A and is trivial to determine the p values.

We didn’t perform the test for PCC as those values are similar to sensitivity values and would not add any additional information. Finally, we have made modifications in the text to show exact p values for CSMF accuracy values of OAA-NBC vs NBC too.”

Q15. A minor point: when discussing the Wilcoxon signed rank......... would this be left out?

REPLY 15: Thank you for pointing this out. It was a typo, and we changed it to “rank two to rank four” in the text. All rank 1 to rank 5 values were used.

**Discussion**

Q16. A number of results are described in the discussion section ................. tables included as appendices as needed?

REPLY 16: We would like to keep these results separate from the main results and actual method as they are not part of the proposed method. Dirichlet distribution based variations of the test set is not the recommended approach in standard text of machine learning; however, researchers in VA studies have used this method for evaluation of algorithms. So, for consistent comparison with the literature we have also performed experiments using Dirichlet distribution. We have also added details of results based on Dirichlet distribution in the Appendix A. Similarly, we would like to keep pre-trained models separate too as all other algorithms have customized training. Pre-trained models actually generate poor results and it is not fair to compare them with the customized model in the Results section.

Q17. In Table 6, it is not clear why ‘HIV/AIDS-related deaths’....... please could the authors clarify?

REPLY 17: Thank you for noting this as this was a typo, we fixed this error in the Table 6.

Q18. It would be pertinent to acknowledge, again, ................. describing “dual physician *assignment based on* *VA data* and clinical diagnostic COD…” would more clearly make the distinction.

REPLY 18: Thank you for pointing this out, we have modified the text as suggested.

Q19. It would be useful to include a paragraph comparing the results .......... If not, what are the differences in the exercises undertaken?

REPLY 19: In terms of the paper pointed out by reviewers on Tariff algorithm, our results show that for PHMRC adult and child, PCCC values remain around 30%  for the first rank (see Appendix A)  and James et al. reported in the range of 22-40% (for only the first rank). Similarly, mean CSMF values in our case remain closer to 70% and their median CSMF values also remain closer to 70%. The main difference, however, is that they have partitioned PHMRC data based on health care experience, and we have used all PHMRC data and a partition of PHRMC based on Indian origin. It is not possible to compare the results exactly due to different partitions.

We have added complete details of the results in the Appendix A, and it should be transparent now in terms of comparison with any paper. Due to many differences in the setup of the experiments (as noted above), it is not possible to write the similarities and differences with all the past studies in one paragraph. This is mitigated by the fact that we have executed all the algorithms and shown all the results in a transparent manner. Thus, individual comparisons with studies will not generate any value in terms of comparisons of results.

Q20. The reporting of cumulative sensitivity as the main measure of agreement is an unusual aspect of this study; acknowledgment of this as a potential limitation would help provide context for comparisons with other similar studies.

REPLY 20: We disagree with the reviewers on this comment. There seems to be some confusion around this concept with reviewers. We have added clarification in the text about the concept as per their earlier comment. We have not introduced any new way of measuring performance of algorithms; in fact the cumulative frequency, cumulative distributions, etc. are common concepts in statistics. It is also common in applied machine learning literature (see above). In the case of top rank prediction (rank 1), results are the same as traditional method of reporting sensitivity, PCCC, CMSF or any other measure. For the next most likely predictions—i.e., rank 2 and onwards, cumulative values just show the sum of previous values. It is a very simple concept; it only provides additional information and does not hide or conceal any results. This is actually the richness of the information in the paper and not the weakness of the paper in any way because earlier researchers have not shown such information. We believe that public health community will only benefit more from such information.

**Minor points**

Q21. In the abstract, the authors write: ................... “The results demonstrate that our approach improves the classification by between 6% and 8% compared with the other algorithms”.

REPLY 21: We have made the change as per your suggestion.

Q22. The Matlab and Agincourt datasets are referred to as DHS – it would be better to refer to them as Health and Demographic Surveillance Sites (HDSS) to prevent confusion between these and the Demographic and Health Surveys.

REPLY 22: Thank you for pointing this out. We changed the text “South African Agincourt Demographic and Health Survey (DHS) dataset , and Bangladeshi Matlab DHS dataset” to “South African Agincourt Demographic Surveillance Sites (HDSS) dataset , and Bangladeshi Matlab HDSS dataset.”

Q23. The article switches from past to present tense a number of times; ....................we would suggest re-writing these passages in past tense.

REPLY 23: Changed

Q24. Probable typo: methods, paragraph 2, line 15 – “datasets” should be “dataset”?

REPLY 24: Changed

Q25. Per normal conventions, please consider adding legends to tables and figures spelling out any acronyms used

REPLY 25: We have carefully reviewed all the tables and figures. We have added description of acronyms for WHO, ICD, COD and VA. For the names of datasets and algorithms, expansions of their acronyms in the tables seem to add lots of redundant information for known items, we have avoided that.
